# Polymeric Frontiers in Next-Generation Energy Storage: Bridging Molecular Design, Multifunctionality, and Device Applications Across Batteries, Supercapacitors, Solid-State Systems, and Beyond

**DOI:** 10.3390/polym17202800

**Published:** 2025-10-20

**Authors:** Akhil Sharma, Sonu Sharma, Monu Sharma, Vikas Sharma, Shivika Sharma, Iyyakkannu Sivanesan

**Affiliations:** 1 School of Bioengineering and Biosciences, Lovely Professional University, Jalandhar 144411, Punjab, India; sharmaakhil177@gmail.com (A.S.); biotech_vikas@rediffmail.com (V.S.); shivikasharma25@gmail.com (S.S.); 2 Baba Nahar Biotech and Research, Bilaspur 174001, Himachal Pradesh, India; sharmasonu70462@gmail.com (S.S.); monusharma311998@gmail.com (M.S.); 3 Department of Environmental Health Science, Human and Eco Care Center, Konkuk University, 1 Hwayang-dong, Gwangjin-gu, Seoul 05029, Republic of Korea

**Keywords:** polymer materials, energy storage, multifunctionality, solid electrolytes, nanocomposites, sustainability, machine learning

## Abstract

Polymer materials have become promising candidates for next-generation energy storage, with structural tunability, multifunctionality, and compatibility with a variety of device platforms. They have a molecular design capable of customizing ion and electron transport routes, integrating redox-active species, and enhancing interfacial stability, surpassing the drawbacks of traditional inorganic systems. New developments have been made in multifunctional polymers that have the ability to combine conductivity, mechanical properties, thermal stability, and self-healing into a single scaffold system, which is useful in battery, supercapacitor, and solid-state applications. By incorporating polymers with carbon nanostructures, ceramics, or two-dimensional materials, hybrid polymer nanocomposites improve electrochemical performance, durability, and mechanical compliance, and the solid polymer electrolytes, as well as artificial solid electrolyte interphases, resolve dendrite growth and safety issues. The multifunctionality also extends to flexibility, stretchability, and miniaturization, which implies that polymers are suitable for use in wearable devices and biomedical devices. At the same time, sustainable polymer innovation focuses on bio-based feedstocks, which can be recycled, and green synthesis pathways. Polymer discovery using artificial intelligence and machine learning is faster than standard methods, predicts structure–property–performance relationships, and can be rationally engineered. Although there are difficulties in stability during long periods, scalability, and trade-offs between indeedness and mechanical endurance, polymers are a promising avenue with regard to dependable, safe, and sustainable power storage. This review presents the molecular strategies, multifunctional uses, and prospects, where polymers are at the center of the next-generation energy technologies.

## 1. Introduction

The use of polymer materials has quickly become one of the leading preferences when it comes to energy storage studies, a landslide shift in the manner in which researchers see the future of power generation, storage, and delivery in future devices. Their essential properties, structural versatility, molecular tunability, and ability to be multifunctional distinguish them from conventional inorganic materials and place them in a unique position to bridge long-standing energy gaps in batteries, supercapacitors, and solid-state systems [1]. Polymeric frontiers’ vision of energy storage is based on their capability to combine molecular engineering with scalable device integration, overcoming limitations of the previous generation of materials and motivating holistic system innovation that meets real-world requirements, such as portable electronics and grid-scale renewables. The polymer materials used in energy storage have a huge spectrum, ranging from molecular design to device-level application. At the molecular scale, the chemist has an unprecedented control over the polymer structure and distribution of functional groups that can be subjected to specific engineering of electron and ion transport routes that are vital for energy harvesting and transfer [2]. Non-rigid crystalline lattices, such as polymers, have the ability to be produced with custom degrees of flexibility, porosity, and conductivity that allow them to be used in thin-film batteries, bendable supercapacitors, and future-generation all-solid systems [3]. This flexibility not only enables the development of a set of customizable energy devices but also leaves the door open to multifunctionality: an interplay between charge storage and self-healing, stretchability, and even a response to the environment. The promised multifunctionality is not just limited to performance improvement but also opens new possibilities of completely new types of devices that are tough, modular, and reconfigurable to new areas of application in the future.

The concept of molecular design in polymeric energy storage is based on a number of scientific requirements. The first is the problem of ion transport: inorganic solids may inhibit dynamic ion movement through the lattice constraints, but the polymers provide a chance to construct segmental dynamics and ion-conducting channels at the molecular level. Second, polymers are capable of accommodating various redox-active species that can include organic molecules, conducting segments, and dopant species, and perform synergistically in battery electrodes and electrode films in supercapacitors [4]. Third, functional additives and nanoinclusions also increase the charge mobility, mechanical integrity, and processability, making a significant jump between the molecular structure and the performance at the device level. Finally, their ability to be functionalized easily and their capacity to produce green and low-energy products give them further sustainability benefits and make them appealing solutions to power sustainability in a carbon-reduced world. Polymeric frontiers are based on multifunctionality [5].

Compared with traditional energy storage materials, which will often trade one property off against another of maximum capacitance versus flexibility or conductivity, polymers can be designed to simultaneously satisfy many of the desired characteristics. As an example, polymer nanocomposites consist of the combination of the strong electron transmission properties of carbon-based nanostructures and the stretchability and self-healing properties of organic matrices, resulting in the development of advanced supercapacitors that do not lose their performance even under the influence of mechanical forces or the surrounding environment. Comparably, solid-state battery polymers also come with the prospect of being both ionically conductive and thermally stable, therefore rendering their usage safe in a wide variety of scenarios [6]. Physical, chemical, and biological functionalities integrated on the same polymer scaffold challenge conventional constraints and establish new standards for energy storage systems to be used in wearable, biomedical, and flexible electronic applications. Understanding what is lacking in traditional materials is important to emphasize the importance of polymers in the quest to store energy in the next generation [7].

Inorganic substances, despite their crystalline perfection, are often brittle, hard to work with, and cannot be used in lightweight, portable, or flexible electronics. They are very hard and restrict the freedom of design to the extent of involving safety risks, such as dendrite axis or catastrophic collapse. Also, the traditional device architectures are ill-equipped to blend disparate functional needs like a high power density and environmental responsiveness, or the incorporation of a self-repair capability. Inorganic matrices do not have the processability or molecular-level customization required to support real system-level innovation, even when it is possible to make multifunctional composites [8]. Polymers confront them directly: their synthetic versatility allows them to be modified on a molecular scale, their softness and stretchability create new application horizons, and their natural versatility creates opportunities to integrate mechanical, electrical, and ionic properties into one material platform. Such a distinct combination of characteristics puts them at the center of ground-shifting strategies in the development of high-energy storage technology [9]. Recent advances in polymer science have triggered the technological path to the deployment of multifunctional devices, starting with the molecular design. Another important breakthrough that was recently announced with the use of polymer materials in battery development was presented by Shi et al. In this paper, a cubic-garnet (Li_7_ La_3_ Zr_2_ O_12_, LLZO) lithium-sulfur battery design based on a gel polymer buffer at the sulfur cathode/LLZO interface was demonstrated. A bilayer LLZO (dense/porous) solid electrolyte was used in the system and allowed a high sulfur loading (5.2 mg cm^−1^) and an initial discharge capacity of 1542 mAh g^−1^ and could be cycled at room temperature and with no external pressure at 80% capacity retention in 265 cycles. The novel polymer interface design was able to provide a solution to critical interfacial instability, which provided a base to stable, high-capacity, and commercially viable garnet lithium-sulfur batteries [10].

**Figure 1:**
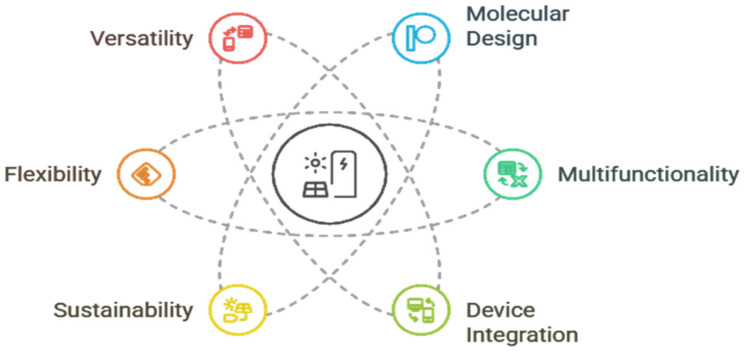
Key attributes of polymer materials contributing to advanced energy storage applications (made by using Napkin software version 0.8.2).

This review starts with setting the principles of polymer materials as energy storage materials, which provide them with a distinct molecular tunability and multifunctionality that sets them apart from more traditional inorganic materials. More work has been performed on more sophisticated molecular engineering approaches where synthetic chemistry and computational chemistry are used to design polymers with desired charge transport and stability properties. Such development of general concepts into specific molecular design provides readers with a solid idea of the material basics of next-generation energy technologies. Figure 1 shows the key polymer properties play a crucial role in enhancing energy storage performance

## 2. Computer-Aided Molecular Engineering Innovations

AI-assisted property optimization and next-generation design strategies have enabled scientists to cross frontiers in materials. This capability to adjust the polymer composition in fine detail at the monomer, nanoparticle, and composite levels is directly translated to performance advantages in energy storage tools. In one example, carbon nanotubes or graphene sheet-based engineered conducting polymers have produced supercapacitors that have record-breaking capacitance and stability, and hybrid polymer electrolytes have made lithium metal an efficient and safe battery [11]. Polymer systems have a particularly rich interfacial chemistry; in addition to being hosts to ion transport and redox reactions, polymer systems provide mechanical damping and structural support, which are important for cycling durability and robust operability. Regardless of such progress, there are still sharp gaps and unaddressed issues. Polymers have weaknesses in chemical stability during long-term service and can be degraded in high-voltage or high-temperature environments [12]. The polymer material interface with other parts of the device, like the electrode electrolyte interface, could bring new complexities in terms of ion movements, electrochemical stability, and mechanical integrity.

The industrial application of fine-tuned polymer systems is a real problem with respect to scaling and reproducibility and requires interdisciplinary solutions in synthesis, process engineering, and computational models. The environmental problem of polymer manufacturing and disposal is also worth continuous consideration, which inspires further study of bio-derived and recyclable polymers that should meet performance and sustainability requirements [13]. To fill these gaps, a complete research agenda is needed, integrating the state-of-the-art characterization, predictive modeling, and holistic engineering of devices to achieve the complete promise of polymeric frontiers in energy storage. In the scientific community, there is a growing agreement on the transformative role of polymers in energy storage, which is motivated by the fact that polymers permeate the dichotomies of the past. Polymers are flexible, tough, accessible, and high-performing, and are closing the gap between molecular complexity and simplicity of the device [14].

Their development in batteries, supercapacitors, and solid-state systems signifies a paradigm shift in which materials science, synthetic chemistry, and electrochemical engineering converge to deliver not marginal benefits but systemic innovation. Now that researchers are going beyond the usual limits and establishing new methods of molecular design and system integration, the polymeric frontier will help to redefine the limits of energy storage and delivery, establishing the stage of an era of next-generation material-powered systems [15]. To conclude, a scientific, dynamic, multidisciplinary, and radically transformative vision has been highlighted in the introduction of polymeric frontiers to next-generation energy storage. Moving beyond traditional constraints and tackling the root causes of the inherent limitations of conventional materials, polymers provide an avenue toward smarter, safer, and more adaptable energy systems through the application of molecular design principles, the embrace of multifunctionality, and confronting the fundamental limitations of traditional materials [16]. Much work will be needed, though, to achieve long-term progress through continuous novelty, research cooperation, and fidelity, yet the trends and potential of polymer studies already signal a new phase that will bring together the gaps in theory and achievement in the pursuit of better energy technologies.

The need to develop new energy storage systems for an advanced energy storage system is increasing in line with the blistering development of portable electronics, renewable energy infrastructure, and battery technology in electric vehicles. This global energy shift and increasing environmental inquiries and constraints of traditional storage apparatuses have stimulated multidisciplinary research in seeking material platforms that can overcome the synergized challenges of performance, sustainability, and scalability [17]. In this context, polymer materials have become a disruptive category with a distinct combination of molecular control, mechanical versatility, and multidimensional capabilities that have never been seen before in inorganic substrates. The frontiers of polymeric energy storage have a very interesting vision: to exploit the peculiarities of the organic molecules, such as their low weight, versatile structure, and compatibility with green synthesis procedures, to create materials and systems that should be better than the existing solutions in all the important criteria [18].

The traditional batteries and supercapacitors that are, in many cases, premised on rigid inorganic crystals are also intrinsically limited by structural inflexibility, mass, and ecological footprint. Although their performance is admirable based on the capacity to store charges or deliver power, they are often undermined in the areas of safety, recyclability, and the capability to respond to new application areas like wearable biomedical devices or roll-to-roll technologies of flexible electronics [19]. This discrepancy between the capabilities of the material and the needs of the system is a central justification of molecular design in contemporary energy storage. Unlike inorganic polymer systems, polymer systems can be designed and engineered at the level of the monomers themselves, allowing the chemical composition, topology, and microstructure to be controlled, unlike before. The optimization of charge transport pathways is made possible by the adjustment of charge transport pathways, electronic and ionic, within the matrix through the tailoring of backbone rigidity, side-chain functionality, and crosslinking density [20]. Consequently, polymers can be engineered to have high ion-exchange functionality and selective redox, thermal, or mechanical stability, which is the main focus of next-generation batteries, solid-state devices, and high-rate supercapacitors.

Recent innovations have shown that conjugated polymers, biopolymers, and composite heterostructures may be used to deal with multiple modalities at the same time. As a case in point, n-type π-conjugated backbones can be engineered at the molecular level, which supports the use of high-rate lithium-ion and sodium-ion batteries [21]. The polymer binders and anodic active materials can enable high efficiency, capacity maintenance, and rate sensitivity. Moreover, the scalable synthesis and low cost, as well as the introduction of electro-polymerizable additives and artificial solid electrolyte interfaces, increase the potential range and operational safety of the devices at the expense of their application [22]. The inherent electronic conductivity and delocalized π-electron systems of π-conjugated polymers provide considerable benefits over the non-conductive binders used in prior studies (e.g., polyvinylidene fluoride, PVDF) in preserving electronic percolation, particularly at high areal loadings. Conventional binders are passive constituents, tending to induce compromised electron paths with an increase in the thickness of the electrode. Contrarily, π-conjugated systems, including polyaniline, polypyrrole, PEDOT and their analogs, create continuous, electronically conductive networks through the composite electrode, and facilitate rapid charge transport when the composite electrode is loaded with large amounts of mass. In addition, they can create smooth surfaces with active substances, which increase the connections between the particles and decrease internal resistance. Recent reports show that electrodes with pie-conjugated polymers as binders have an excellent retention capacity, rate and a low impedance at higher areal loadings than conventional binder systems with such strong percolative networks and synergistic electronic contributions from the polymer matrix itself [23]. These features are not just scholarly innovations but are true developments toward more practical next-generation power systems capable of comfortably fitting new device architectures.

The basics of polymer molecular design are determined, and at this point, the discussion concentrates on the concept of multifunctionality. In this section, the way polymers are designed to satisfy the demands of the attributes of electrical conductivity, mechanical integrity, thermal stability, and self-healing characteristics is discussed for the development of flexible and durable energy storage devices.

## 3. Molecular Engineering of Advanced Polymer Systems

**Figure 2:**
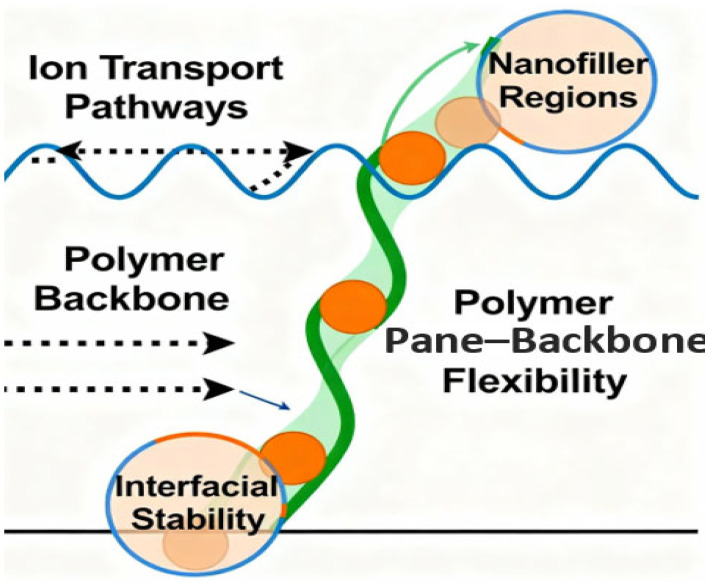
Schematic representation of polymer membrane showing ion transport pathways (wavy blue zig-zag lines), regions with nanofiller particles (orange circles), and important multifunctional areas such as polymer backbone, pane-backbone flexibility, and sites of interfacial stability (circled areas). The arrows indicate how ions move through the membrane and highlight where flexibility as well as stability are improved.

The molecular engineering of superior polymer systems to store energy is an interdisciplinary innovation in synthetic chemistry, computational modeling, and convergence at the interface of synthetic chemistry with functional materials science that can transform the limits of the field. The scientific community is also concentrating on the methods of engineering polymer backbones and side chains to maximally enhance the conductivity, stability, and multifunctional use in next-generation batteries, supercapacitors, and solid-state devices [22]. The paradigm shift has gone beyond traditional empirical design and currently exploits AI-aided modeling, high-throughput computational strategies, and green synthetic methodologies, and is the age of rational material engineering, which used to be considered impossible. The modern methods of designing polymer molecules start with a realization that the backbone structure of a polymer defines its electronic characteristics, efficiency of transporting ions, and general stability under running conditions [24] (Figure 2).

Chemists can design polymers with predictable performance due to the precise synthetic control of monomer selection, sequence distribution, and chain architecture. An example is the addition of π-conjugated systems to polymer backbones to increase electron delocalization and redox activity, which is required in energy storage programs in lithium-ion batteries and supercapacitors [25]. Customized side-chain capabilities, in turn, tune ionic conductivity, solubility, and interfacial adhesion, which, in solid-state electrolytes and flexible device matrices, is a decisive lever toward performance optimization. In addition to conventional wet chemistry, AI-aided and computational technologies have changed the speed at which polymers are discovered and optimized several times [26]. The high-throughput in silico screening of candidate materials with optimal energy storage properties using machine learning (ML) models that are trained on large datasets of polymer structures, physical properties, and device performance metrics is made possible. They are algorithmic approaches that find latent relationships between molecular structures and emergent behaviors that can be used to steer synthesis systems to generate structures that can deliver increased ionic mobility, dielectric breakdown strength, and mechanical compliance [27].

Transfer learning and multitask neural networks are ML-based methods that allow researchers to work with polymer systems that are too complex and multiscale. They not only optimize individual property domains but also contribute to the balancing of several, and usually conflicting, functional requirements. Importantly, computational screening based on data and stochastic breakdown simulations has aided in the prediction of dielectric breakdown processes and energy densities in polymer-based composites, and ML frameworks have been constructed that include variables (including dielectric constants, sizes, and contents of fillers) and their combinations to predict and experimentally validate the process [28]. These high-throughput methodologies play a very important role in the rational design of polymer composites, in particular in areas where trial-and-error synthesis would have been resource-intensive or systematic. Simulation and laboratory results have supported these in silico paradigms, in which the gap between the theoretical design and experimental data has been reduced, to bring high-energy polymer capacitors and solid-state battery electrolytes closer to the goal of innovation. Another aspect of molecular engineering is the need to have a green synthesis process and a green source of materials [29]. Recent studies focus on the design of bio-based polymers, i.e., the use of renewable monomers and environmentally friendly processing methods to minimize the carbon footprint and toxicological effects of new energy storage materials [30]. The use of naturally occurring macromolecules, catalytic chain-growth processes, and recyclable polymer matrices has allowed the creation of friendly energy devices, which resonate with the growing concern of a circular material economy and regulation in high-end manufacturing industries.

Yoon et al. [31] introduced an explainable ML model, which used optical absorbance spectra to make quick predictions of the electrical conductivity of doped conjugated polymers, and tested their predictions experimentally on brand new material systems. These case studies are examples of the practical combination of computational prediction and laboratory validation of modern polymer material development [31]. The interpretation of ML models to predict the polyimide dielectric constant was performed by He et al. [32]. The researchers synthesized three new polyimides based on the model, and the experimental results indicated that the deviation between the ML model and predictions was less than three percent, indicating the strength of the ML model in electronic materials discovery [32]. Bradford et al. [33] trained an Arrhenius-informed ML model to make predictions of the ionic conductivity of polymer electrolytes. The model was trained using a large set of experimental results and was shown to be very accurate in comparison with laboratory measurements in the case of numerous polymer structures, making it useful in a real-world situation of selecting electrolytes in solid polymers [33]. In 2024, Lin et al. created a interpretable ML model for predicting polymer thermal conductivity (based on GBDT) using molecular features, which was validated in subsequent synthesis, and so it provides a physical understanding and reliable experimental validation [34].

The integration of green chemistry concepts with molecular engineering is an important step toward responsible innovation that will guarantee the scalability of polymeric energy technologies in the future and their universal availability across the world. In spite of such developments, there are still significant gaps related to the logical design of polymers that are to be used in high-temperature, multifunctional, or fully bio-based applications. The lack of standardized datasets, small coverage of extreme-use cases, and gaps in mapping structure–property combinations prevent many machine learning models from achieving high levels of generalizability and predictive performance in special energy storage applications [30]. The thermal and chemical stability of bio-based polymers is not yet everywhere, similar to that of petroleum-based analogs, and property integration at multiscale (i.e., high ionic conductivity and mechanical strength, thermal durability, etc.) is not always without trade-offs, which are not readily modellable in existing computational models. These loopholes are even made more complicated due to the difficulties in modeling complex interface phenomena, including the electrode-to-electrolyte compatibility and degradation routes in cyclic or extreme operating conditions [35]. To overcome these weaknesses, scientists are spending time on constructing complete, high-quality databases, constructing interpretable ML models, and coming up with experimental guidelines to prove the models’ predictions and increase the size of structural exploration.

The use of convolutional neural networks to analyze nanoparticle fillers, reinforcement learning to optimize silicon nanoparticles and the polymer binder, and support vector machines to predict the lifetime of marine biodegradable composites are just a few examples of how molecular engineering and AI can work together to achieve discoveries in energy storage materials [36]. Such collaboration not only allows the design of polymers with a specific functional profile but also empowers predictive maintenance, process control, and early failure intervention of deployed storage systems. Finally, the new approaches to synthetic control coupled with computational intelligence and green chemistry are revolutionizing the field of molecular engineering of innovative polymer systems to store energy [37]. Controlled manipulation of the polymer backbone and side-chain designs, with inputs of machine learning and simulation, has widened the scope of accessible properties of ionic conductivity, mechanical flexibility, dielectric strength, and operational safety of flexible and multifunctional energy platforms. Although rational design still has some gaps, particularly in extreme environmental contexts and complete material sustainability, there is a steady improvement in the data collection, model formulation, and validation that are closing the gaps [38]. The combination of AI-based molecular prediction, high-throughput simulation, and experimentally sound synthesis has become the new paradigm in the field, where the future of the next-generation energy storage systems is only bounded by imagination and cross-disciplinary cooperation.

Innovative molecular engineering of novel polymeric energy storage systems is one of the emerging critical frontiers in materials science, synthesizing synthetic chemistry, computational innovation, and green methods to build better devices than are possible today. This part elaborates on approaches to designing polymer backbone and side-chain architectures to store energy, the applications of machine learning, new functionalization approaches, and challenges and gaps in design, with a concentration on scientific novelty and global understanding [5]. The basis of molecular engineering is the specific control of the polymer backbone. The chemical structure of the backbone is the major determinant of the electronic properties of the polymer, mechanical stability, and ion mobility.

The mobility of charge carriers, redox reactivity, and thermal stability are directly dependent on different backbone chemistries that can be conjugated to zero-systems through aliphatic chains with polar functional groups. As an example, conjugated polymers having both single and double bonds are good sources of extended pi-electron delocalization, which mainly boosts electrical conductivity and allows redox reactions essential to battery electrodes and pseudocapacitive materials [39]. Nonetheless, these polymers have to be processible and flexible, and therefore it is common that polymers undergo copolymerization with non-conjugated sections or that side chains that control both solubility and interchain separation are introduced. Equally important is the concept of side-chain engineering to control the performance of polymers. The side chains not only affect the solubility and processability but also the ion transport and mechanical characteristics [40]. The introduction of ionic or polar pendant groups enhances ionic conduction, which is important in solid polymer electrolytes, and the ion exchange reaction is fast without the destruction of polymer stability. The presence of long alkyl side chains confers mechanical flexibility and may disrupt crystallinity to increase amorphous regions that are conducive to ion movement. Additionally, the side-chain architecture, such as length, branching, and density, can be tuned precisely, giving control of thermal and chemical stability, which are critical for polymers exposed to extreme electrochemical conditions or high temperatures [41]. Synergistic backbone and side-chain design thereby forms multifunctional polymers to meet application-specific requirements.

Contemporary molecular engineering ceases to depend on the classical laboratory synthesis and chemical intuition. However, computational techniques, in particular, machine learning (ML), have turned polymer design into a science of data. ML algorithms, including graph neural networks, support vector machines, and Bayesian optimization, learn the relationships between the structures and properties of polymers with large-scale datasets to understand the complex structure–property relationship [42]. It is a method that allows the testing of polymer candidates virtually within a few hours with predicted and high values in the parameters of conductivity, mechanical strength, thermal endurance, and electrochemical stability of the polymer, which are vital in energy storage systems. ML-assisted design involves several steps. The first step is to build detailed polymer databases with accurate models of molecular structures and related physicochemical properties. The second step is training predictive models on such data, capturing relationships, as well as predicting the behavior of polymers based on the desired behavior. The third step involves utilizing virtual screening and optimization algorithms to generate new polymer structures that match the desired behavior [43].

Particularly, more recent progress in multifidelity learning enables the integration of experimental and computational measurements, and hence enables maximum accuracy of a model even when there is a small amount of high-quality experimental measurements. These computational methods have hastened discovery, especially of multifunctional polymers, wherein trial and error methods are strictly forbidden by the time requirements of classical methods. As an example, ML models have been used to predict the ideal filler materials and polymer matrix composition to improve the dielectric breakdown strength and energy density of polymer nanocomposites used in the production of flexible capacitors. With this development, microstructural motifs and side-chain functionalities can be identified, and they can work together to maximize charge storage without compromising flexibility or longevity [44]. Moreover, ML enables a trade-off between conflicting properties, including the maximization of ionic conductivity and the maintenance of mechanical strength, which is a key factor of solid polymer electrolytes used in batteries. The molecular design is closely related to green synthesis and sustainability concerns. The new trend towards bio-based polymers based on renewable feeds demands novel molecular architectures that do not reduce but, in fact, improve performance and have a minimal environmental impact. They include the use of biodegradable moieties, enzymatic polymerization techniques, and low-toxicity solvents and catalysts [45]. The special attention in regard to this is that such approaches demand a close molecular design in ensuring that biopolymer materials have similar ion transportation capacities, chemical stability, and mechanical qualities to the synthetic polymers.

Research in molecular engineering is emerging as a nascent field, but projections show that bio-derived polymers are poised to bridge the divide with promising electrochemical performance. Despite all these developments, there is still a lot left to be filled and a lot to be done, especially in the rational design of high-temperature and multifunctional polymer systems [46]. Anisotropic temperature stability requires polymer backbones and side chains that are not thermoxidatively degradable, not ionically conductive, and maintain mechanical integrity. This makes the design of such materials complicated, with the improvement of one property affecting the other. The available state-of-the-art ML models are constrained by the lack of quality data on polymer behavior under extreme conditions, which limits their ability to make predictions. In addition, the chemical architecture and mesoscale morphology interactions that have a powerful impact on properties such as ion conduction paths and mechanical damping are not well represented in existing computational systems [47].

**Figure 3:**
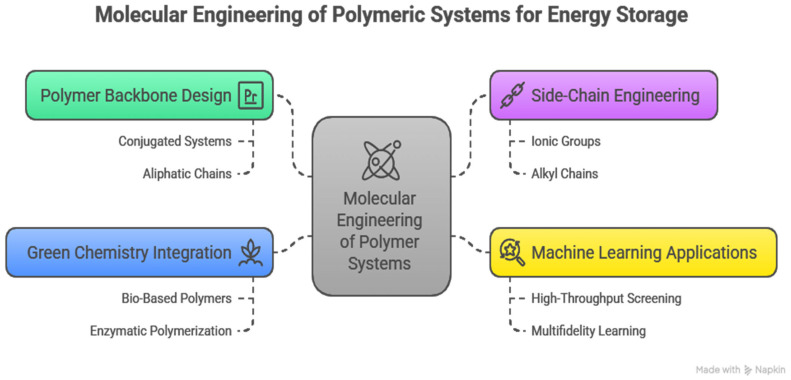
Molecular engineering of polymer systems for energy storage (made by using Napkin software version 0.8.2).

The other important gap is connected to the creation and production of entirely bio-based polymers that offer multifunctional properties and can be compared to those based on petroleum. These polymers should incorporate biodegradability, electrochemical performance, and processability, which is a daunting trifecta that is yet to be achieved [43]. A mechanistic understanding of matters and innovative methods and strategies at the molecular level, in association with AI-based design, are necessary to overcome these challenges, as they lead to a concept of polymers that meet the demands of the future energy environment and minimize the performance of the devices. The main strategies for molecular engineering of polymeric systems are illustrated in Figure 3.

## 4. Multifunctionality: Beyond Single Performance Metrics

The multifunctionality of polymer materials as energy storage materials is transforming the paradigm of optimization of individual measures of performance, including conductivity or mechanical strength. The changing requirements for the next-generation energy apparatuses, especially batteries, supercapacitors, and flexible solid-state systems, require the incorporation of various functional characteristics into a single material platform. This integration entails the synthesis of electrical conductivity, mechanical durability, thermal stability, and advanced self-healing functionality, which are crucial for promoting the efficiency, durability, and safety of devices [48]. The fact that these properties can be combined in one polymer material is a scientific breakthrough, enabling the creation of a device that can resist mechanical deformation, thermal stress, and long-term electrochemical cycling, broadening their application to the challenging demands of wearable electronics, as well as central energy storage in the grid.

Electrical conductivity underlines the existence of energy-storing polymers since it is primary in terms of transporting charge effectively to guarantee a high power density. Nonetheless, polymers have long had low conductivity compared to inorganic metals and ceramics. To address it, molecular engineering methods include conductive moieties like conjugated backbones and doping mechanisms, which boost the circulation of electrons and do not affect flexibility [49]. At the same time, mechanical robustness is essential, which allows the polymers to resist fractures, fatigue, and deformation under repetitive loads, which is necessary especially when used in flexible and wearable devices that are therefore subjected to bending and stretching. Researchers develop materials that do not compromise mechanical strength to increase conductivity through molecular tailoring, such as crosslinking strategies, the incorporation of elastomeric segments, and creation of nanocomposites [50]. This balance is needed because any device switching and charge–discharge processes are bound to cause both mechanical and electrochemical stresses. These properties are complemented by thermal stability, which ensures the integrity and performance of polymers at different operating temperatures. Energy equipment is commonly used under varying thermal conditions due to the exposure of the equipment to the environment or inherent heating of the equipment in Joule heating conditions during high-rate charging and discharging [51].

Thermally stable backbones, flame-retardant side chains, and crosslinked networks in polymers allow the polymers to withstand higher temperatures without losing their conductivity and mechanical properties. This property makes the devices safer and more resilient to counterbalance the disaster failure modes associated with material breakdown. In addition, the use of self-healing features brings in a paradigm shift in longevity improvement [52]. Dynamic covalent bonds or reversible supramolecular interactions used as polymers can heal microcracks or chemical damage independently and thus increase the life of devices and minimize their maintenance expenses. This capability is especially appreciated in the novel wearable or implantable energy storage systems where service is restricted. Polymeric nanocomposites are more positively multifunctional with the addition of two-dimensional (2D) and three-dimensional (3D) nanomaterials, including graphene, metal–organic frameworks (MOFs), and MXenes, which have shown synergies in various fields [53].

Graphene, an outstanding electrical conductor, has mechanical strength and a specific surface area, and can act as a conductive scaffold besides enhancing structural strengthening at the nanoscale. Its incorporation in polymer matrices leads to high capacitance of the composite, cyclic stability, and mechanical durability. MOFs also provide porosity and adjustable chemistry, which increases the rates of ion and electrolyte accessibility that are essential for a high rate of charge storage [54]. The more recent group of 2D transition metal carbides and nitrides, called MXenes, has integrated layered structures with metallic conductivity as well as hydrophilic surfaces, leading to efficient ion diffusion and high electrode–electrolyte interactions. The incorporation of MXenes has shown an exceptional enhancement of electrochemical performance, such as increased capacitance, charge–discharge rates, and cycle life, and, at the same time, anti-corrosion and self-passivating properties [55]. This type of multifunctionality of nanocomposites is revolutionary in actual devices. In the case of batteries and supercapacitors, the polymer matrices supported by these new nanomaterials connect conduction orders and mechanical strength, which polymers or fillers do not have.

In the case of flexible solid-state systems, such composites exhibit mechanical compliance and do not reduce ionic or electrical conductivity. In addition, these polymer nanocomposites with multifunctional characteristics enable environmental resilience against humidity, temperature changes, and mechanical deformations that are essential for business viability. The composite method further allows functional gradient customization, with various parts of a device playing different functions such as mechanical support, ion transport, or charge collection, being a philosophy of materials-by-design. In spite of these developments, significant gaps and issues still exist, especially with respect to modular trade-offs of multifunctionality [56]. The process of combining several properties in one material may be associated with complicated interdependencies, where an improvement in one of the properties may adversely affect another. As an example, mechanical strength can be enhanced by increasing the density of crosslinks, which can lower ionic mobility and conductivity. In the same way, the addition of high levels of nanofillers to increase conductivity may affect the processability and the flexibility of the polymer as well as form interfacial flaws, which compromise reliability.

**Figure 4:**
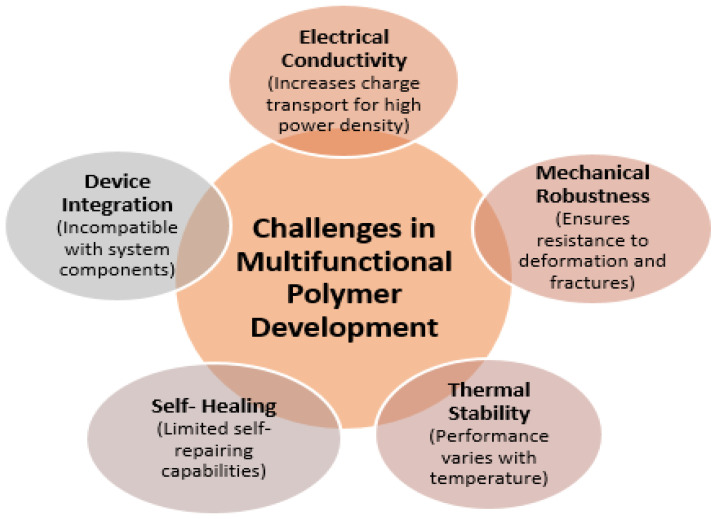
Key challenges in developing multifunctional polymers, including conductivity, robustness, stability, self-healing, and device integration.

Multifunctional polymer systems also often have challenges in integration at the device level because of their incompatibility with other components of the system in terms of mechanical or thermal behavior, resulting in stress buildup and possible points of failure (Figure 4). These trade-offs require a strict knowledge of structure–property–process interaction at several length scales, between molecules and device structures. It is urgently required to have frameworks that systematize the optimization of the balance between conflicting functional properties, taking advantage of advanced characterization techniques (including in situ spectroscopy and tomography) and predictive modelling [57]. Moreover, the creation of standardized testing procedures to measure multifunctionality as a whole will speed up the process of screening and optimization of material. The other difficulty is the scalability and reproducibility of multifunctional polymer composites.

The production of manufacturing processes that guarantee homogeneous nanomaterial dispersions and unified polymer deformation is essential to convert the prototypes that are working in the laboratory into commercially viable products. Fluctuations in material batches/processing conditions will cause inconsistency in the performance of the device, which will interfere with industrial adoption [58]. The development of manufacturing methods, including controlled 3D printing, electrospinning, and roll-to-roll coating, is promising, but it needs to be optimized further to be used with multifunctional polymer systems.

Having insights into the multifunctional nature of polymer materials, this section discusses how these materials can be incorporated into real-life energy storage solutions. The following transition emphases the translation of molecular and multifunctional properties into the key components of batteries, supercapacitors, and solid-state systems, which reflects the evolution of matter to the device.

## 5. Polymer Materials for Advanced Energy Storage Devices

### 5.1. Polymeric Innovations in Batteries

Polymers’ innovation in a wide range of device platforms, particularly batteries, has brought revolutionary changes in energy storage technology that are essential to meet the increasing needs of portable electronics, electric cars, and renewable energy sources. The core of this innovation lies in the innovations of polymeric and solid electrodes, solid electrolytes, and artificial solid electrolyte interphases (SEIs) that are considered to be central in determining the performance, safety, and scalability of devices. It is the basis of lithium-ion, sodium-ion, and multivalent battery systems, and current research efforts are pushing the limits of polymer integration to increase ionic conductivity, mechanical robustness, interfaces, and long-term cyclability [59]. Conductive polymers have become indispensable elements of battery electrodes because of the attributes of electronic conduction, flexibility, and chemical versatility. Representative systems are polyaniline (PANI), polypyrrole (Ppy), or poly(3,4-ethylenedioxythiophene) (PEDOT); all of these systems promote the rapid transfer of electrons and serve as binders that ensure the integrity of the structure during the process of lithiation and delithiation. Such conducting polymers are commonly used in conjunction with inorganic active materials or carbonaceous nanostructures to create polymer/metal and polymer/nanocarbon hybrids to address the issue of volume swelling, mechanical breakdown, and electronic disconnection that afflict traditional electrodes [60]. As an example, polymer composites with graphene or carbon nanotubes have been shown to have superior conductivity networks with a higher mechanical buffering capacity, allowing sustained high-rate operation and better cycle life in lithium-ion and emerging sodium-ion batteries. The trend of increasing the polymer scope to multivalent batteries (the use of ions like Mg^2+^, Zn^2+^, or Al^3+^) only reinforces this point of view.

The dense charge layering and slow kinetics of multivalent ions drive the need for polymeric electrodes and electrolytes, which can allow dynamic charge transport processes without a loss of mechanical stability or chemical compatibility. Customized ion recognition sites on polymers and flexible structures enable the diffusion of multivalent ions, which is considered the bottleneck of ionic transport and interphase formation between electrodes. These advances bring into focus the versatile nature of polymer materials that can be molecularly engineered to suit the unique chemical requirements of different battery systems [61].

Solid polymer electrolytes exhibit much higher thermal stability, lack leakage hazards, and inhibit lithium dendrite growth, another severe failure mechanism of metal anode batteries, as compared to their liquid counterparts. Polyethylene oxide (PEO)-based electrolytes, polycarbonates, and poly(vinylidene fluoride) (PVDF) derivatives have been largely investigated because they have good ionic conductivity and mechanical characteristics [62]. The chemistry of adding nanoscale fillers such as ceramic oxides or sulfides to these polymers forms composite electrolytes with high mechanical strength and an ionic transport pathway, overcoming the conductivity versus mechanical reliability trade-off inherent in pure polymer electrolytes. The basic concept of ion transport in these solid polymer electrolytes is that the segmental chain dynamics at the glass transition temperature (Tg) control ion transport. Reducing Tg by innovations in polymer chemistry to add plasticizers has thus been the key to enhancing ionic mobility. Also, polymer engineering approaches like crosslinking and copolymerization trade off conductivity and mechanical strength, which allows the use of electrolytes that retain their functionality under operating conditions and deformation [63]. Electrolyte durability and mechanisms of contact between electrodes and interfaces at the molecular and morphological scale are further enhanced with the assistance of mechanical engineering, such as the creation of interpenetrating polymer networks and graded interfaces. These properties are crucial to achieving solid-state batteries that have a high energy density, long cycle life, sufficient flexibility to meet the evolving flexible electronic requirements, and can be used with a wide range of cathode materials. Another aspect of polymer innovation that is important to battery stability and performance is artificial solid electrolyte interphases (SEIs) [64].

Artificial SEIs are engineered by a polymer coating or interlayers on the surface of electrodes and have several purposes: to prevent parasitic reactions between electrode and electrolyte, to transport ions, and to reduce dendritic growth on metal electrodes, leading to short-circuit and capacity loss. Highly developed polymeric SEIs have been developed, possessing characteristics of ionic transportability, mechanical elasticity, and chemical inertness. SEI layers made of a dynamic or self-healing polymer can accommodate changes in volume during cycling and stay continuously covered, ensuring that they do not grow harmful dendritic structures [65]. New directions include the in situ polymerization of electrode surfaces or the use of functional additives to be polymerized during electrochemical cycling that will confer flexibility and durability to SEIs under complex battery conditions. Still, there are pronounced loopholes that obstruct the large-scale adoption of polymer elements in the device platforms. The ability to perform under long-term cycling and other severe environmental conditions (i.e., temperature changes, exposure to electrolytes, etc.) is a key concern [66]. A large portion of polymer electrolytes and artificial SEI materials degrade or, in other words, mechanically fail, which restricts the life and safe operating limits of batteries.

The mechanical softness of polymers that makes them be able to be flexible can also cause a risk of dendrite penetration or mechanical breakage when high current densities are applied, and designing advanced polymers that will strengthen polymer layers without negatively impacting ion transport is necessary. The other significant challenge is scalability. High-performance polymer electrolyte and electrode synthesis and processing methods need to be cost-effective and allow production at scale [67]. At industrial scales, a homogenous dispersion of nanofillers, consistent polymer molecular weights, and structure are hard to control. In addition, uniform protocols for fabrication and quality control processes are still under development to allow the attainment of reliable integration with the variety of electrode materials and battery formats.

**Figure 5:**
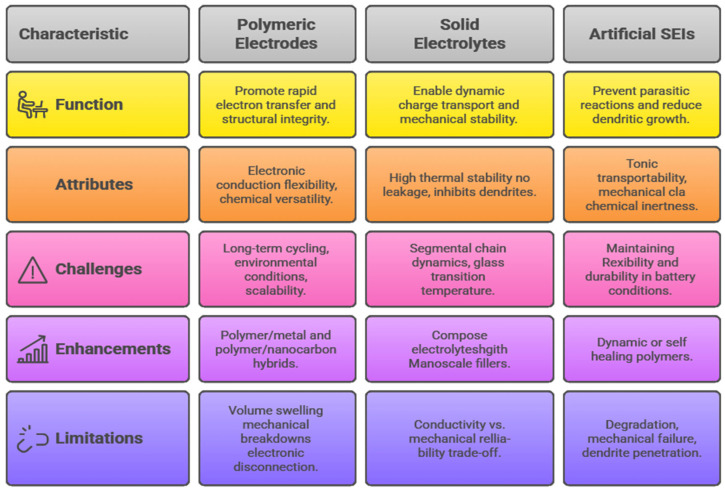
Comparative overview of polymeric electrodes, solid electrolytes, and artificial SEIs in advancing battery technology (made by using Napkin software version 0.8.2).

The interfacial issues of the polymer components and the other battery materials also limit performance and life. Stresses, delamination, and loss of contact caused by mismatches in thermal expansion coefficients, chemical potentials, and the mechanical behavior of the polymer at polymer–electrode surfaces impair ion transport and accelerate degradation [68]. The solutions to these problems require a comprehensive view of the interface chemistry, novel modes of characterization, and novel engineering solutions to these problems, including graded interfaces, functionalized polymer surfaces, and organic–inorganic hybrids. Figure 5 shows the comparative overview of polymeric electrodes, solid electrolytes, and artificial SEIs in advancing battery technology.

### 5.2. Polymer Materials in Supercapacitors and Pseudocapacitors

Supercapacitors and pseudocapacitors are crucial elements in the energy storage environment, offering exceptional benefits in charge storage dynamics, power density, and cycle life. Their operation is inherently connected with molecular design strategies and material structures, which contribute to increased pseudocapacitance, a faradaic charge storage process that is independent of the electrostatic processes that determine the operation of electric double-layer capacitors (EDLCs) [69]. Within the framework of next-generation energy storage devices, particularly polymer-based systems, insights into and the development of charge storage mechanisms; the design of hybrid architectures of conjugated polymers, nanocomposites, and metal–organic frameworks (MOFs); and overcoming the longstanding issue of a disconnect between the energy and power density gap are all still at the leading edge of high-impact materials science.

Pseudocapacitors’ charge storage processes are largely determined by the presence of fast and reversible faradaic reactions that take place on or at the electrode surface, rather than the simple physical adsorption of EDLCs. Such faradaic reactions are redox, ion intercalation, and electrosorption reactions, which allow the pseudocapacitors to attain multiple oxidation states and attain much higher energy densities than other supercapacitors [70]. Combined capacitive and battery-like properties make the pseudocapacitive behavior stand out, with an almost linear charge–potential relationship, rapid kinetics, and excellent cyclability. Transition metal oxides (e.g., RuO_2_, MnO_2_, NiO), conducting polymers (e.g., polyaniline (PANI), polypyrrole (Ppy), and poly(3,4-ethylenedioxythiophene) (PEDOT), and emerging 2D materials (e.g., MXenes) have high values of pseudocapacitance because these materials can participate in redox reactions at the surface or near the surface. The design of polymer materials with increased pseudocapacitance relies on the electronic structure and morphology of the material to promote efficient ion diffusion and electron transfer [71].

Reversible redox reactions that involve the doping and de-doping of conductors are conducted on polymers, changing their oxidation level and charge density. Both the polymer backbone and side chain functionality affect the electrical conductivity, as well as ion accessibility and mechanical strength throughout any volumetric changes that occur during redox cycling [72]. To achieve high pseudocapacitance, a low molecular weight, and sufficiently spaced electrochemical potential windows, polymers, including PANI and Ppy, are preferred because they offer a large number of accessible redox sites. On the other hand, polymers with a higher molecular weight, such as PEDOT, although they have a lower theoretical capacitance, have better cyclic stability and flexibility because of their extraordinarily high ionic mobility and resistance to structural strain caused by charge–discharge cycles. The trade-offs of electrical, chemical, and mechanical characteristics in the design of polymers are critical to support the long cycle life and high capacitance of the polymers during the actual operation periods [73]. Hybrid Architectures improve the performance of pseudocapacitors by mixing polymers with complementary nanomaterials that extend the surface area, enhance conductivity, and structural stability.

Nanocomposites based on polymers conjugated with carbonaceous nanotechnical materials, including graphene, carbon nanotubes (CNTs), and activated carbon, take advantage of the high surface area and good electron transport mechanisms provided by these nano-additives. This integration enables the quick transfer of charges and enhances electrochemically active sites to greatly increase capacitance and energy density [74]. In addition, metal–organic frameworks (MOFs) implement porous, crystalline, tunable chemical functionalities in ion transport and accessible redox centers, which are useful as templates or fillers in a polymer matrix. MOF composites have synergistic benefits and are able to incorporate the mechanical flexibility and redox characteristics of polymers with the structured porosity and catalytic actions of MOFs, thereby maximizing both pseudocapacitive and electrical double-layer effects. The potential of the hybrid approach can also be seen in the intercalation pseudocapacitance mechanism that is especially prominent in layered 2D materials, including MXenes. MXenes have great electrical conductivity with hydrophilic functional groups that allow the efficient ion intercalation without disrupting the lattice [75]. It is combined with conducting polymers to create composite electrodes that combine electrical double-layer and faradaic pseudocapacitive storage and achieve volumetric capacitances up to 1500 F/cm^3^. The outcome of these hybrid systems is the faster charge–discharge rate, the high rates of operation, and the mechanical robustness that is essential for the commercialization of these systems in flexible and high-power energy storage devices [76].

Although significant strides have been made to date, the fundamental issue of overcoming the intrinsic energy–power density gap has not been addressed. Pseudocapacitors provide a better energy density compared to EDLCs, but tend not to be as good as batteries. The simultaneous high energy and power densities required, coupled with the need to ensure long-term stability, require innovative designs of polymers in which the molecular architecture is maximally designed to provide the highest accessibility of redox sites, minimal resistance to charge transfer, and augmented ion diffusion pathways [69]. Redox cycling of many present-day polymers has trade-offs between capacitance and cycle life because of volumetric changes caused by redox cycling that cause mechanical stress and eventual degradation. To address this, molecular designs need to include flexible backbones, self-healing functionality, and strong nanocomposite structures to counter structural fatigue.

In terms of flexible devices, wearable devices, solid-state systems, sustainable polymer systems, and next-generation computational aids to energy storage and electronic devices are fast advancing in their constantly increasing needs to converge in the flexibility, sustainability, and guided material design. Polymers are playing a central role in this revolution, and they allow breakthrough functionalities of flexibility, stretchability, self-repair, and miniaturization that are critical to wearable and solid-state devices [4]. At the same time, a sustainable and green production process for polymers corresponds to the emergent materials that meet the needs of ecology, whereas AI-based computational chemistry and discovery optimization speed up the innovation and optimization of devices. It is an integrated scientific account of these frontiers as a group, explaining how they support one another, what they face as they develop, and how we can use them to be the first generation of energy storage technology.

The new application opportunities associated with wearable health monitors, flexible displays, and implantable biosensors are materials based on polymers with the ability to behave mechanical compliance and dynamic adaptability in biological tissues. Flexibility and stretchability are central mechanical properties such that devices can go through repeated bending, twisting, and stretching without mechanical exhaustion and a loss of functionality [77]. Polymers, with their natural, versatile control of chemical composition and building blocks, meet these demands far better than conventional inorganic materials. An example of biodegradable and mechanically resilient bio-elastomers that are important for transient electronics and soft biomedical devices is bio-elastomers like polyglyceryl sebacate (PGS) and poly(octamethylene citrate) (POC). Chemical and physical crosslinking strategies increase elasticity, which facilitates stretchability that maintains electrical characteristics and guarantees intimate but non-invasive contact with soft tissues. In addition, self-healing polymers are a breakthrough in terms of the reliability and durability of devices [7]. Self-healing processes use dynamic covalent bonds, supramolecular interactions, or reversible physical crosslinking that allows spontaneous healing of microcracks or other damage caused by mechanical deformation or by chemical degradation. Such properties improve the life of a device, decrease maintenance costs, and improve the safety of the user, particularly users of implantable medical electronics and wearable sensors under repetitive stress.

Self-healing dynamical hydrogel composites with conductive properties are an example of a multifunctional polymer that combines mechanical stability and high electronic functionality. Soft robotics are at the forefront of innovation and energy harvesting technologies. Besides mechanical strength, the miniaturization of flexible polymeric energy storage devices demands processing and fabrication technological innovations [78]. Electrospinning, 3D printing, and roll-to-roll coating methods enable the fine control of polymer morphology, can be made into thin and lightweight films and meshes, and can be incorporated into compact devices via the architecture. These miniaturized polymer materials have large surface area-to-volume ratios needed for energy density maximization in supercapacitors and micro-batteries. Electrospinning is known as an easy, flexible, and scalable technique of producing large-surface-area nanofibers of a polymer that can be utilized in high energy storage devices. Although the reuse of labor-scale electrospinning devices is economically feasible in prototyping and preliminary research, industrial-scale production will have to consider a number of economic considerations, including throughput, solvent recovery, uniformity, and environmental safety. Recent improvement in multi-jet, needleless, and roll-to-roll electrospinning technologies have greatly enhanced the level of productivity and minimized labor expenses. Mass production of nanofibers with controlled morphology has been shown to be possible in industry, but solvent usage and ability to effectively collect solvents are also factors that affects the overall cost benefit ratio. The literature indicates that electrospinning may enter the realm of commercial viability when used on a large scale with closed-loop solvents and continuous processing configurations and that this approach can provide a trade-off between quality of the product, scalability, and financial feasibility [79]. Molecular design is necessary to enhance polymers’ intrinsic properties, including ionic conductivity, electrochemical stability, and adhesion properties, to fit in the advanced fabrication methods and to ensure that the processes remain effective at smaller scales.

The sustainability requirement is becoming more and more popular in polymer innovation. The environmental impacts of the production of polymers, their consumption, and disposal are being questioned, and there is a serious effort to develop bio-based polymers, renewable feedstocks, greener solvent systems, and recyclable materials. Cellulosic and lignin-based polymers have also demonstrated potential both in terms of degradability and end-of-life recyclability, and provide both mechanical and electronic performance suitable for energy storage uses [14]. As an example, cellulose nanofibril films have been investigated as flexible substrates and separators with great tensile strength, gas barrier properties, and biocompatibility. Green manufacturing plans are oriented to decrease the quantities of toxic byproducts, decrease the consumption of energy, and work in solvent-free or aqueous-phase manufacturing.

Enzymatic polymerization, photopolymerization, and microwave-assisted synthesis have been applied, and these methods help to make the production routes cleaner and more efficient. These long-term strategies are oriented toward the alignment of high-performance polymer production with regulatory requirements and environmental responsibility. Nonetheless, difficulties in the scale-up of bio-based polymers still exist, especially in terms of chemical homogeneity, structural regulation, and functional equality between bio-based polymers and petrochemical-based counterparts [80]. The solutions to these gaps include multidisciplinary activities such as polymer chemistry, process engineering, and life cycle assessments to make sure that sustainability promises do not harm the performance of devices.

### 5.3. Sustainable and Green Polymer Materials for Energy Storage

The fast-changing paradigm of energy storage and electronic devices requires more and more integration of flexibility, sustainability, and intelligent material design. Polymer materials are central to this change and have made possible the breakthrough functionalities of flexibility, stretchability, self-repair, and miniaturization that are required in wearable and solid-state devices. At the same time, sustainable synthesis and green production of polymers match new materials with environmental demands, and AI-based computational chemistry and data-driven discovery help to speed up the pace of innovations and optimization of devices [81]. This comprehensive scientific account considers these fronts in their entirety, explaining their interdependency, changing issues, and opportunities in the face of the next generation of energy storage technologies.

The new areas of wearable health monitors, flexible displays, and implantable biosensors demand the use of polymeric materials that mimic the mechanical compliance and adaptability characteristics of biological tissues. Mechanical properties: Flexibility and stretchability are the basic mechanical properties enabling certain devices to bend, twist, and stretch repeatedly without mechanical failure or loss of functionality. Polymers, whose chemical composition and architecture can be tuned and which have a wide range of architectures, can meet these needs much more effectively than traditional inorganic materials. Examples of bio-elastomers with a balance between biodegradability and mechanical stability are polyglyceryl sebacate (PGS) and poly(octamethylene citrate) (POC), which offer transient electronics and soft biomedical applications [82]. Practical and chemical crosslinking methods contribute to the increase in elasticity, which allows stretchability to maintain electrical functionality and contact with soft tissues that is intimate but not invasive. In addition, the creation of self-healing polymers is a breakthrough in terms of the reliability and durability of devices.

Dynamical covalent bonds, supramolecular interactions, or reversible physical cross-links in self-healing polymers are used to spontaneously repair microcracks, other types of mechanical deformation, or chemically degraded damage. Such properties extend the life of devices, lower maintenance expenses, and improve the safety of users, an extremely important aspect of implantable medical electronics and wearable sensors that could be subjected to high-frequency stress. Besides mechanical strength, the miniaturization of flexible polymeric energy storage devices requires innovative processes and fabrication technology [83]. Methods such as electrospinning, 3D printing, and roll-to-roll coating provide good control of polymer morphologies, create thin and lightweight films and meshes, and can be incorporated into compact device designs. These miniaturized polymer materials provide the large surface area–volume ratios required for the energy density maximization of supercapacitors and micro-batteries. Molecular design is used to optimize polymers’ intrinsic properties, including ionic conductivity, electrochemical stability, and adhesion qualities, so that they can be used in such advanced fabrication techniques to guarantee that they will work at smaller scales.

## 6. Emerging Frontiers: AI, Computational Chemistry, and Data-Driven Discovery

In the complicated environment of polymer design and device integration, there has emerged a paradigm shift in the use of computational tools and especially artificial intelligence (AI) and machine learning (ML). These tools provide a systematic analysis of large amounts of structure–property–performance data that can be used to predict and optimize polymer functionalities, which otherwise would be unattainable through trial and error using only experimental data alone. Machine learning (ML) and artificial intelligence (AI) are no longer considered as computational aids, but rather as the driving forces of polymer innovation. It is these techniques that are now used to push the process of rational discovery, prediction, and validation to shrink experimental timescales once measured in years to weeks. In polymer science, AI algorithms can identify structure–property–performance relationships using huge datasets that are made up of molecular descriptors, synthesis parameters, and device outputs. As an example, to predict ionic conductivity and dielectric constants, polymer graphs can be directly transformed into graph neural networks (GNNs) and transformer-based models and be accurately predicted [84]. In the meantime, monomer combinations that maximize conductivity in solid electrolyte stability trade-offs are autonomously suggested by reinforcement learning (RL) frameworks.

Virtual screening with high throughput using ML has already been used to discover candidate backbones with optimized ion transport pathways of lithium polymer electrolytes and predict polymer chain motifs that enable a high dielectric strength. Simultaneously, generative adversarial networks (GANs) and Bayesian optimization algorithms develop new polymer structures with desired mechanical capabilities and redox potentials. Such predictions with AI are then verified with automated synthesis as well as in situ characterization and generate a closed-loop design pipeline, which continuously enhances model fidelity [85].

ML algorithms can help to screen molecular candidates, optimize the processing parameters, as well as clarify the degradation pathways, thereby reducing the innovation cycles and improving reproducibility [84]. As an example, AI-based molecular dynamics simulations offer a more faithful prediction of the ion migration pathways in solid polymer electrolytes to inform the molecular engineering of polymers with greater ionic conduction and mechanical strength. Equally, generative models synthesize polymer structures with enhanced mechanical flexibility to be worn and at the same time balance electrochemical performance needs. The discovery made through data discovery improves the current knowledge about the degradation mechanisms and mechanical breakdown, allowing predictive maintenance and lifetime optimization of flexible energy devices. However, these computing boundaries are significantly limited [85].

**Figure 6:**
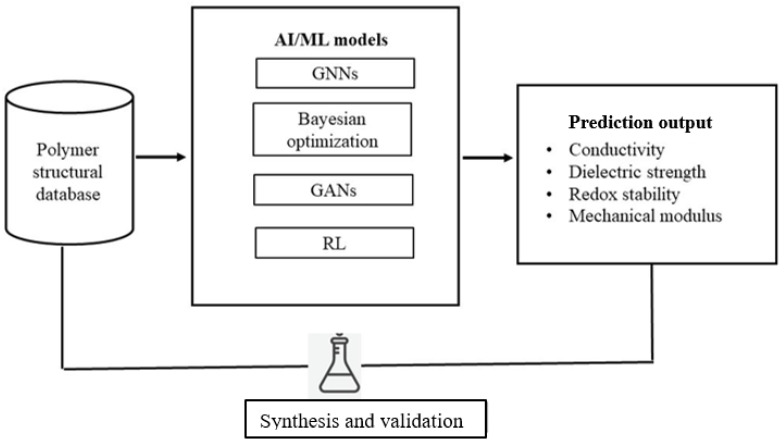
Schematic representation of a data-driven workflow illustrating how machine learning models predict polymer properties and are refined through synthesis and validation feedback.

The accessibility of a high-quality, full dataset is a major bottleneck that limits model accuracy and applicability between polymer classes and application conditions. Most datasets are not characterized using standardized characterization protocols, and, therefore, inter-laboratory comparisons are challenging. The disparity between the atomic scale models and the macroscopic level in the operation of devices poses more computational issues that demand multiscale modeling paradigms [27]. The development of the explainability and interpretability of AI models is essential to user trust and regulatory acceptance of AI models, particularly safety-oriented biomedical devices. Schematic representation of a data-driven workflow illustrating machine learning model prediction, synthesis, and validation of polymer properties (Figure 6), alongside an overview of polymer systems used in supercapacitors and next-generation energy storage (Table 1).

In the complicated environment of polymer design and device integration, computational chemistry, artificial intelligence (AI), and machine learning (ML) have led to a paradigm shift. These are used to quantitatively examine large amounts of structure–property–performance data, thereby predicting and optimizing polymer functions that were previously unattainable through purely experimental trial-and-error methodology. ML algorithms can help to filter molecular candidates, optimize processing parameters, clarify degradation pathways, reduce the duration of innovation, and improve reproducibility [40]. As an illustration, a molecular dynamics simulation using AI is more likely to predict ion transport pathways in solid polymer electrolytes in a more faithful manner, informing the molecular engineering of polymers with an increased ionic conductivity and mechanical strength.

**Figure 7:**
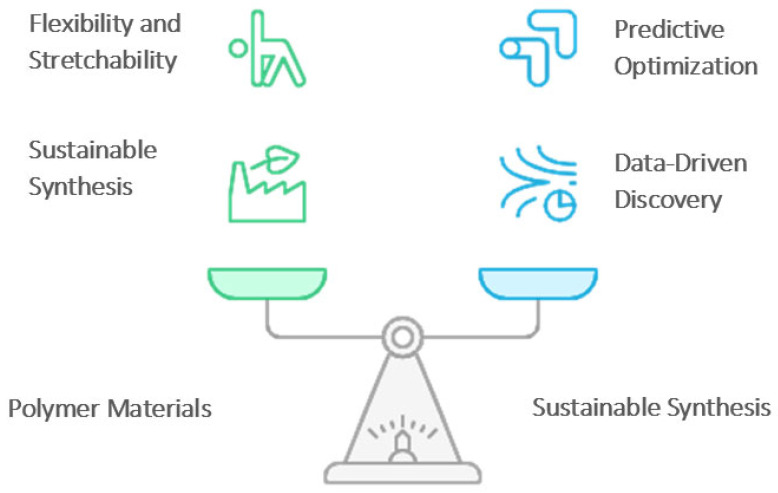
Synergistic roles of polymer materials and computational tools in advancing energy storage innovation (made by using Napkin software version 0.8.2).

Equally, generative models produce polymer scaffolds characterized by optimized mechanical dexterity to wearable tasks and trade-offs in electrochemical functioning. This is because data-driven discovery is increasing knowledge of degradation kinetics and mechanical failure modes to compute predictive maintenance and lifetime optimization of flexible energy devices. There are, however, significant limitations to these computational frontiers. Access to large, high-quality datasets is also a major bottleneck and limits the accuracy and transferability of the models across types of polymers and to different application settings. A large number of data sets do not have standardized characterization protocols, and thus an inter-laboratory comparison is not possible. Overcoming the discrepancies between small-scale simulations and the large-scale behavior of a device is an additional computational problem that needs multiscale modeling frameworks [82]. The adaptability of AI models in the interpretation and explainability of AI models is essential for gaining user confidence and regulatory approval, particularly in safety-sensitive biomedical technologies. Figure 7 represents the synergistic role of polymer material and computational tools in advancing energy storage innovation.

## 7. Sustainable Polymer Materials and Green Manufacturing

The need to be sustainable is increasingly being felt in polymer innovation [86]. The ecological footprint of polymer manufacturing, processing, and recycling is currently under review, leading to concerted effort on bio-based polymers, renewable feedstocks, greener solvent systems, and recyclables. Cellulose and other naturally available precursors to polymers have demonstrated potential in not only being degradable and circuit to end-of-life recyclability but also providing mechanical and electronic performance, allowing their use in energy storage applications. As an example, cellulose nanofibril films are studied as flexible substrates and separators, which have high tensile strength and gas barrier properties and are biocompatible [87].

Green manufacturing policies aim at minimizing toxic byproducts, minimizing the use of energy, and turning to solvent-free processes. Enzymatic polymerization, photopolymerization, and microwave-assisted synthesis are implemented to achieve cleaner and efficient production pathways. Such sustainable approaches are expected to balance high-performance polymer production, regulatory requirements, and environmental responsibility [88,89]. Nonetheless, the scaling of bio-based polymers remains a problem, especially in the attainment of chemical uniformity, structural control, and functional parity between bio-based polymers and those made of petrochemicals. To overcome these gaps, there are multi-disciplinary actions that include polymer chemistry, process engineering, and life cycle assessments to ensure that the sustainability promises are not achieved at the expense of the device performance [90,91].

## 8. Future Perspectives, Research Gaps, and Innovation Roadmap

Polymer materials have the potential to revolutionize energy storage in the future, with the ability to be multifunctional, scalable, and embedded into a variety of platforms in devices. Their development is guided toward the facilitation of high power and energy densities, reliability, and cost-effectiveness. This is possible by designing hybrid polymer architectures consisting of conductivity, stability, and the ability to be mechanically flexible to provide greater functionality in batteries, supercapacitors, and solid-state systems without hurting manufacturability. One major issue is the need to have a balance between high charge–discharge rates and energy storage over the long term. Developments in controlled polymer molecular design and conductive nanomaterial composites are a possibility, although the optimization of ionic/electronic transport, interfacial stability, and polymer morphology at various scales is still important. The other issue is long-term reliability because polymers are subject to chemical degradation, mechanical fatigue, and interfacial failure when used in repeated cycling. Innovations are being made in developing self-healing polymers, mechanically compliant environments, and stable dynamic interfaces that do not allow capacity loss and structural disintegration.

Hybrid systems that incorporate organic polymers with inorganic fillers or nanostructures can improve on the limitations of individual materials by combining the flexibility of polymers and ionic conductivity of ceramics. New technologies in polymer chemistry, along with dynamic covalent bonding, supramolecular assembly, and sequence-controlled polymerization, allow the development of networks with responsive ion transport, mechanical strength, and flexibility in solid-state devices. Future developments involve interdisciplinary work that involves chemistry, materials science, computational modeling, and engineering. The predictive tools for structure–property–performance relationships will require advanced characterization methods and machine learning software for quick discovery and optimization. Another important aspect is sustainability through environmentally friendly synthesis, recyclability, and life cycle analysis. The polymeric energy storage innovation roadmap, in general, focuses on multifunctionality, durability, scalable manufacturing, and sustainable design. These issues can be mitigated through the integration of science and technologies that have enabled polymer-based systems to form the backbone of the future generation of energy storage that is high-performance and environmentally friendly.

## 9. Conclusions: The Path Forward

Polymers have become central materials in the emerging energy storage technologies of the next generation as a unique interface between molecular design and polymerization for multifunctional use in batteries, supercapacitors, and solid-state devices. They have outstanding structural and chemical tunability, which allows the integration of electric conductivity, mechanical strength, thermal stability, and adaptive functions in monolithic material systems. Combined with scalable and sustainable manufacturing, polymers are the base of flexible, wearable, and high-performance energy devices. In this paper, the essence of molecular design is highlighted in order to make sure that the structures of polymer backbones and side-chain functionalities are optimized to reach better ion transport, redox activity, and interfacial stability. One of the design requirements that will be emphasized is multifunctionality, and polymers with the highest energy and power densities and improved durability will be developed using self-healing and mechanical compliance. Artificial interfaces between solid electrolytes and new hybrid nanocomposites, solid electrolytes, and artificial interfaces are made to work synergistically to increase ionic conductivity, mechanical strength, and interfacial stability and develop high-performance and long-lasting and efficient energy storage systems. Polymer integration between devices can allow the energy storage capacity to surpass the growing requirement of IoT-enabled wearables, flexible electronics, and sustainable large-scale systems. Other obstacles have included high power and energy densities at the same time, long-term stability under varying working conditions, and multifunctional polymer characteristics to suit hybrid and solid-state batteries. They need to be overcome with interdisciplinary research using polymer chemistry, computational modeling, data-driven discovery, and environmentally friendly engineering. The next roadmap does not merely encompass material growth on its own, but the incorporation of the devices as well as scaled fabrication in accordance with environmental sustainability. Lastly, the present discussion confirms the role of polymers as central participants in energy storage of the future and they will unlock new energies through the convergence of molecular design, multi-functionality and device synergy to catalyze transformative contributions in smart and flexible and sustainable energy strategies.

## Figures and Tables

**Table 1:** Overview of the polymer systems used in supercapacitors and next-generation energy storage.

Section	Key Concepts and Mechanisms	Materials/Strategies	Challenges	Future Prospects
Supercapacitors and Pseudocapacitors	– EDLCs: electrostatic storage – Pseudocapacitors: Faradaic redox, ion intercalation, and electrosorption reactions – Linear charge–potential relation, high cyclability	– Transition metal oxides (RuO_2_, MnO_2_, and NiO) – Conducting polymers (PANI, Ppy, and PEDOT) – 2D materials (MXenes)	– Energy–power density gap – Volumetric changes during cycling – Stability vs. capacitance trade-offs	– Molecular design for more redox sites – Self-healing, flexible polymers – Hybrid EDLC + pseudocapacitive storage
Polymer Redox Mechanisms	– Doping/de-doping alters oxidation and the charge density – Conductivity depends on the backbone and side-chain design	– PANI and Ppy → high capacitance – PEDOT → stability and flexibility	– Structural strain from redox cycling – Trade-off: capacitance vs. cycle life	– Low-molecular-weight polymers for high redox activity – High MW for durability and flexibility
Hybrid Architectures	– Combine polymers with nanomaterials – Synergistic conductivity, surface area, redox activity	– CNTs, graphene, activated carbon composites – MOF–polymer composites—MXene–polymer systems (1500 F/cm^3^)	– Mechanical stress during charge/discharge – Stability at high rates	– Layered nanostructures for ion intercalation – Multifunctional nanocomposites
Flexible and Wearable Systems	– Flexibility, stretchability, and self-repair are crucial for the biocompatibility for implantable devices	– Bio-elastomers (PGS and POC) – Self-healing polymers – Conductive hydrogels	– Mechanical fatigue – Maintaining conductivity under deformation	– Smart biomedical devices – Soft robotics – Miniaturized, printed energy devices
Sustainable Polymer Materials	– Bio-based, recyclable, green synthesis	– Cellulose and lignin-based polymers – CNF films for separators/substrates – Enzymatic and photopolymerization routes	– Scale-up limitations – Chemical homogeneity issues	– Life cycle assessment integration – Green solvent systems – Regulatory alignment
AI and Computational Chemistry	– AI/ML for predicting structural properties – Molecular dynamics for ion transport – Generative models for polymer design	– AI-based simulations for SPEs – Data-driven degradation pathway analysis	– Lack of high-quality datasets and multiscale modeling challenges – Model interpretability issues	– Faster discovery cycles and predictive maintenance – Data standardization and integration

## Data Availability

The data are available within this article.
